# Deferring Postoperative Follow‑Up Visits up to 4 Weeks After Uneventful Cataract Surgery in a Tertiary Level Eye Hospital: Protocol for a Prospective, Quantitative, Experimental Control Study

**DOI:** 10.2196/48616

**Published:** 2023-08-31

**Authors:** Sanket Parajuli, Parami Dhakhwa, Rabindra Adhikary

**Affiliations:** 1 Reiyukai Eiko Masunaga Eye Hospital Kavrepalanchowk Nepal; 2 Seva Foundation Kathmandu Nepal

**Keywords:** cataract surgery, operational research, SICS, small-incision cataract surgery, postoperative visits, cataract, follow-up visit, postoperative care, surgery

## Abstract

**Background:**

Routine examination after cataract surgery, including a refraction test 4 to 6 weeks after surgery, is mandatory in most hospitals. In recent years, there has been growing interest in exploring alternative approaches to postoperative follow-up in cataract surgery patients due to the increasing number of cataract surgeries being performed, the limited availability of health care resources, and the need to optimize the use of health care services.

**Objective:**

We aim to compare postoperative visual outcomes after a day 0 examination in patients with 2 follow‑ups, one on day 7 and other on day 30, and patients with a single ophthalmic follow‑up between days 25 to 30.

**Methods:**

A prospective, quantitative, experimental control study will be carried out in Reiyukai Eiko Masunaga Eye Hospital, located in Banepa, Kavrepalanchok, Nepal. All patients undergoing cataract surgery meeting the inclusion and exclusion criteria irrespective of the type of surgery (small-incision cataract surgery or phacoemulsification) will be included in the study. The patients will be randomly assigned to 1 of 2 groups. Patients in group 1 will be examined on day 1, day 7, and day 30, whereas patients in group 2 will be examined on day 1 and once between days 25 to 30. The minimum clinically important difference (MCID) in our study will be set according to the improvement in the Snellen visual acuity chart.

**Results:**

The study is expected to be completed within 6 to 8 months from the start of the project. Data analysis and report writing will be carried out in a 2-month period. Best-corrected visual acuity will be compared between the 2 groups to determine if the MCID is achieved. The cost-effectiveness of the new approach will also be analyzed.

**Conclusions:**

We aim to conclude that we can safely defer the 1-week postoperative follow-up visit in patients undergoing uncomplicated cataract surgery and that, moreover, we can reduce the patient load at the hospital and decrease patient expenses by decreasing the frequency of hospital visits.

**International Registered Report Identifier (IRRID):**

PRR1-10.2196/48616

## Introduction

### Background

Cataract is the leading cause of blindness and visual impairment in lower- and middle-income countries [1,2]. Surgery is the only available treatment that is highly cost-effective and capable of significantly improving visual function [3,4]. Cataract surgery often achieves an excellent visual outcome with low rates of complication [5,6]. Routine examination after cataract surgery, including a refraction test a few weeks after surgery, is mandatory in most hospitals [7,8].

Limburg et al [9] investigated the benefits of postoperative follow-up in India and concluded that visual acuity at hospital discharge was not representative of visual acuity several weeks after surgery [10]. However, a study by Congdon et al [11] concluded that early vision assessment of all patients and a follow-up assessment only for patients who return to the clinic without prompting were adequate to ensure the quality of cataract surgical services. This is again countered by a study by Gupta et al [12], who presented evidence of substantial changes between visual acuity on the first postoperative day and at subsequent follow-up. Postoperative complications and uncorrected refractive errors are 2 major issues that lead to poor visual outcomes [5,13-16]. Both issues can be wholly addressed by promoting and ensuring postoperative follow-up at the designated time [17-20]. As per the National Programme of Control of Blindness, Vision 2020: Right to Sight, the recommended postoperative follow‑up visit schedule is a first follow‑up on the first postoperative day by the surgeon (mandatory), a second follow‑up between day 7 and day 10, and a third follow‑up (with refractive correction) between day 30 and day 45 [21]. Saeed et al [22] and Tinley et al [23] concluded that postoperative follow‑up visits can be safely deferred up to 2 weeks after cataract surgery, thereby enhancing the efficiency of day care units. Meltzer et al [24] performed a study using data from the PRECOG (Prospective Review of Early Cataract Outcomes and Grading) trial and reported that visual acuity immediately after cataract surgery was highly correlated with visual acuity after 40 days, suggesting that for the purposes of quality assessment, follow‑up of all patients is not needed. A study by Deshpande et al [25] concluded that visual outcomes after uneventful cataract surgery were comparable between patients followed up on days 3 to 7 and days 25 to 30 in comparison to patients directly followed up on days 25 to 30. Additionally, it was observed that the first follow‑up visit could be safely deferred until 4 weeks after uneventful cataract surgery, including phacoemulsification or small-incision cataract surgery (SICS), in patients with no preexisting ocular or systemic comorbidity [25]. The purpose of this study is to evaluate, from a safety perspective, altering the standard postoperative management guidelines after cataract surgery so that the first follow‑up visit at 1 week is deferred in patients with no significant preexisting ocular or systemic comorbidity and no intraoperative surgical complications or immediate postoperative complications at the time of discharge from the hospital.

### General Objective

The general objective is to reduce follow-up visits in cataract surgery patients.

### Specific Objectives

The specific objectives are (1) to compare postoperative visual outcomes after a day 0 examination in patients with either 2 follow‑up visits, one on day 7 and the other on day 30, or 1 ophthalmic follow‑up visit between day 25 and day 30; (2) to assess the safety of deferring the first follow‑up visit at 1 week; and (3) to perform a cost-benefit analysis of the intervention.

## Methods

### Study Design, Setting, and Participants

This will be a prospective, quantitative, experimental, noninferiority control study of health services use. Selected patients undergoing cataract surgery at Reiyukai Eiko Masunaga Eye Hospital irrespective of the type of surgery (SICS or phacoemulsification) will be included in the study.

All enrolled patients will undergo an examination on the first postoperative day conducted by an ophthalmologist who is unaware of the patient’s group assignment. Subsequently, the patient will proceed to the counselor’s office, where a unique identification number will be generated and the patient will be randomized into either group 1 or group 2.

In group 1, patients will be instructed to attend follow-up visits at 1 week and 1 month postoperatively. In group 2, patients will be instructed to attend a follow-up visit at 1 month postoperatively only. During the visit, the patients will receive detailed guidance on the appropriate use of medications and be educated about the signs and symptoms they should be vigilant for. The specific date of the postoperative visit will also be communicated.

At each follow-up visit, the patient will directly visit the counselor’s office. Upon arrival, they will be provided with a blank sheet of paper containing the unique identification number assigned to them on the first postoperative day. Visual acuity testing will be conducted by a blinded optometrist, while slit lamp biomicroscopy will be performed by a blinded ophthalmologist. Before leaving the hospital, the patient will return the paper to the counselor, who will then enter the necessary information into the patient’s file. 

### Inclusion Criteria

Patients undergoing uncomplicated cataract surgery at Reiyukai Eiko Masunaga Eye Hospital irrespective of the type of surgery (SICS or phacoemulsification) will be included in the study.

### Exclusion Criteria

Patients will be excluded if (1) there are complications during the surgery that warrant more frequent follow-up visits (eg, premature entry, posterior capsular rupture, zonular dialysis, postoperative hyphema, toxic anterior segment syndrome, or postoperative fibrin in the anterior chamber); (2) the surgery is for complicated cataract, traumatic cataract, ocular hypertension, or if the patient has drug allergies; (3) the patients has a history of diseases that might affect postoperative visual outcomes, such as uveitis, glaucoma, viral keratitis, corneal scarring, hypertensive retinopathy, diabetic retinopathy, central retinal vein occlusion, branched retinal vein occlusion, or age-related macular degeneration; (4) the patient is unwilling to participate or does not provide written consent; and (5) the patient misses the follow-up visits.

### Sample Size and Sampling Technique

Sample size is calculated as follows: 2[Zα + Z1 – β] 2 × (sd2/d2) = 2 [1.96 + 1.645 ] 2 × [(0.1125)2/(0.025)2] = 263 for one group, where reference mean values for postoperative visual outcomes are taken from the study conducted by Deshpande et al [25]. The number of participants in intervention group will be 263 and the number of participants in the control (reference) group will be 263. We will use a random sampling technique and computer-generated random numbers to assign patients to the intervention or control group. We generated randomized numbers with a commonly used method [26]. The assignment will be done by the counsellor on the first postoperative day. The researchers will be masked as to randomization at all postoperative visits.

### Data Collection Technique and Tools

A proforma will be developed for data collection. A questionnaire will be prepared; counselors will ask the questions and the information will be recorded in a register. This information will be transferred to an Excel (Microsoft Corp) sheet on a daily basis.

### Plan for Supervision and Monitoring

The study will be carried out by a team of investigators. The principal investigator will provide orientation for the involved staff 1 week prior to participant enrollment. The issues that might arise during the course of data collection will be resolved by mutual discussion between the investigators. The study will be supervised remotely by the Seva team.

### Plan for Data Management and Analysis

Study records that identify the patients will be kept confidential, as required by law. All records pertaining to the patients’ involvement in this research study will be stored in a locked file cabinet in the hospital. A case number will indicate the patients’ identity on these records. This information will only be accessible to the authors.

### Expected Outcome of the Research Results

We expect to determine whether we can defer the 1-week cataract surgery follow-up visit of patients to the hospital.

### Primary Outcome

Visual acuity measurements serve as the most important primary outcome. This includes assessing the level of visual improvement or stability achieved by patients during the 4-week follow-up period. Visual acuity outcomes can provide insights into the effectiveness of deferring follow-up visits for achieving comparable visual outcomes to the standard practice of early postoperative visits.

### Secondary Outcome

The results of the cost-benefit analysis can be considered a secondary outcome. Conducting a cost-effectiveness analysis allows for the assessment of the economic implications and potential cost savings associated with deferring postoperative follow-up visits. This outcome provides insights into the financial feasibility and efficiency of implementing the deferred follow-up strategy.

We will perform a cost-benefit analysis to compare the economic status of the intervention group and active control group. For this we have to collect financial data on direct and indirect costs. Data on direct costs will be retrieved from the hospital record system, because this is the money paid directly by patients for their treatment after the surgery is recorded, including payments for consultation, medicine, and investigation (if required) at each follow-up visit.

Data on indirect costs, that is, costs incurred to the patient in the process of coming to the follow-up visit, include the following: (1) lost productivity costs (how much money the patient could have earned if the patient hadn’t come to the hospital follow-up visit), (2) transportation costs (the cost incurred while travelling), and (3) companion costs (patients will definitely have a companion, for whom we also have to calculate both costs above (lost productivity and transportation). Data on indirect costs will be obtained from interviews with the patients. We will calculate the average cost per visit, which will ultimately reveal the costs and benefits of the new approach, because the suggested new regimen will eliminate one follow-up visit.

### Plan for Use of Research Findings

Research findings will be disseminated through the Reiyukai Eiko Masunaga Eye Hospital newsletter. Donors will be also informed. Results will be published in high-impact journals for better coverage.

### Ethical Considerations

#### Ethical Approval

The Declaration of Helsinki will be adequately addressed. Ethical approval for this study has been obtained from the ethical review board of the Nepal Health Research Council (428/2022 P).

#### Consent

Participants will be provided with detailed information about the study, including the deferral of postoperative visits, as well as potential risks, benefits, and alternatives. A written informed consent form will be provided to all participants.

#### Privacy and Confidentiality

Study data will be collected and stored in a manner that ensures the anonymity or deidentification of participants. Personally identifiable information such as names, addresses, and social security numbers will be removed from the data set. Each participant will be assigned a unique identifier that is used instead of their personal information.

## Results

More than 526 participants will be enrolled in the study, with a minimum of 263 assigned to the intervention group (deferred follow-up visits) and a minimum of 263 to the comparison group (standard practice of early postoperative follow-up visits). The sample will have diverse demographics, ensuring a representative population.

Visual acuity measurements will be obtained for both groups during the follow-up period. We will be able to statistically compute whether the deferred follow-up visit group demonstrates comparable visual acuity outcomes to the comparison group. This will be inferred if there are no statistically significant differences between the two groups in terms of visual acuity outcomes (*P*>.05).

Among the participants in the deferred follow-up visit group, the number of patients who experience postoperative complications during the 4-week follow-up period will be compared with the group with the 1-week follow up visit.

We will also determine if participants in the deferred follow-up visit group report high levels of satisfaction with the extended recovery period and reduced travel burden. The number of participants who express that deferring follow-up visits provided them with convenience and comfort during the recovery phase will be mentioned.

We will determine if reduction in early postoperative visits allows health care providers to allocate their time and expertise more efficiently, focusing on patients with immediate needs or complex postoperative care requirements.

## Discussion

### Expected Findings

The proposed study will investigate the feasibility and potential benefits of deferring postoperative follow-up visits up to 4 weeks after uneventful cataract surgery in a tertiary level eye hospital in Kavrepalanchok.

Deferring postoperative follow-up visits will allow patients to recover in the familiar environment of their homes, reducing the need for immediate travel and associated costs. This can have a positive impact on patient satisfaction and comfort during the recovery period. By avoiding unnecessary trips to the hospital, patients may experience reduced anxiety and stress, leading to an improved overall experience.

By spacing out follow-up visits, health care providers can optimize resource allocation within the tertiary level eye hospital. This approach will allow health care personnel to allocate their time and expertise more efficiently, focusing on patients who require immediate attention or complex postoperative care. Moreover, it can potentially reduce the burden on health care facilities, as fewer appointments are scheduled during the early postoperative period.

Deferring follow-up visits may also improve access to care, particularly for patients residing in remote areas. Traveling to health care facilities shortly after surgery can be challenging, especially for individuals with limited mobility or limited access to transportation. By extending the follow-up period, patients from distant locations can receive necessary care without the immediate need for travel, increasing the likelihood of their adherence to follow-up appointments.

One major concern related to deferring follow-up visits is the potential delay in detecting postoperative complications. Early postoperative visits allow health care providers to closely monitor patients for any signs of infection, inflammation, or other adverse events. When extending the follow-up period, it is crucial to educate patients regarding the signs and symptoms of postoperative complications so that they can visit the hospital, if required, on time. Patients should receive comprehensive information about self-care, potential warning signs, and instructions on when to seek medical attention. This will empower patients to actively participate in their own recovery and promote a sense of responsibility for their eye health.

### Limitations and Challenges

There are potential challenges associated with deferring postoperative follow-up visits. It is important to carefully consider the selection criteria for eligible patients to ensure that only those with a low risk of complications are included in the study. Additionally, proper communication channels must be established to ensure patients have access to health care providers in case of emergency concerns or complications during the extended follow-up period.

### Conclusion

The proposed study on deferring postoperative follow-up visits up to 4 weeks after uneventful cataract surgery in a tertiary level eye hospital in Kavrepalanchok will offer several potential benefits for patients, health care providers, and health care facilities. By prioritizing patient convenience, optimizing resource use, and ensuring appropriate support and education, this approach has the potential to improve access to care and overall patient satisfaction. However, careful consideration should be given to the selection criteria and establishment of effective communication channels to address potential challenges and ensure patient safety. The findings of this study will contribute valuable insights to the delivery of eye care services and may have implications for similar settings.

## Abbreviations

PRECOG: Prospective Review of Early Cataract Outcomes and Grading
SICS: small-incision cataract surgery

## Data Availability

The data sets generated during and/or analyzed during this study are available from the corresponding author on reasonable request.
